# Selection for Reduced Fear of Humans Changes Brain and Cerebellum Size in Red Junglefowl in Line with Effects of Chicken Domestication

**DOI:** 10.3390/brainsci13070988

**Published:** 2023-06-23

**Authors:** Johanna Gjøen, Felipe Cunha, Per Jensen

**Affiliations:** AVIAN Behavioural Physiology and Genomics Group, IFM Biology, Linköping University, 58183 Linköping, Sweden; johanna.gjoen@liu.se (J.G.); felipebrcunha@gmail.com (F.C.)

**Keywords:** domestication, chicken, cerebellum

## Abstract

A central part of the domestication syndrome is a reduction in relative brain size. In chickens, it has previously been shown that domesticated birds have smaller relative brain mass, but larger relative mass of cerebellum, compared to their ancestors, the Red Junglefowl. It has been suggested that tameness may drive the domestication syndrome, so we examined the relationship between brain characteristics and tameness in 31 Red Junglefowl from lines divergently selected during ten generations for tameness. Our focus was on the whole brain, cerebellum, and the remainder of the brain. We used the isotropic fractionator technique to estimate the total number of cells in the cerebellum and differentiate between neurons and non-neuronal cells. We stained the cell nuclei with DAPI and performed cell counting using a fluorescence microscope. NeuN immunostaining was used to identify neurons. The absolute and relative masses of the brains and their regions were determined through weighing. Our analysis revealed that birds selected for low fear of humans (LF) had smaller relative brain mass compared to those selected for high fear of humans (HF). Sex had a significant impact only on the absolute size of the cerebellum, not its relative size. These findings support the notion that selection for increased tameness leads to an enlargement of the relative size of cerebellum in chickens consistent with comparisons of domesticated and ancestral chickens. Surprisingly, the HF birds had a higher density of neurons in the cerebellum compared to the LF line, despite having a smaller cerebellum overall. These findings highlight the intricate relationship between brain structure and behavior in the context of domestication.

## 1. Introduction

Animal domestication has been defined as the process whereby populations of animals change genetically and phenotypically in response to the selection pressure associated with a life under human supervision [1,2,3]. This process is associated with a suite of phenotypic alterations, common across species, often referred to as the domestication syndrome [4,5,6,7,8]. One of the prominent features of this syndrome is a general reduction in relative brain mass in domesticated species compared to their wild ancestors.

Chickens were first domesticated about 8000 years ago from the Red Junglefowl (Gallus gallus), native to Southeast Asia [9]. However, recent analysis has indicated that the domestication may have happened considerably later [10]. Since then, chickens have been selectively bred for various traits, such as meat and egg production, and are now spread worldwide. Comparing contemporary domesticated chickens with ancestral Red Junglefowl, a range of differences have been reported in line with the domestication syndrome, such as alterations of brain mass and composition as well as changes to their social behaviour [11,12,13].

Domesticated chickens typically have a larger cerebellum relative to the total brain mass than Red Junglefowl (RJF), despite an overall smaller brain relative to body size [11]. The cause of this difference is not fully understood, partly because the function of the cerebellum relative to other brain parts is still not clear.

The cerebellum, located in the posterior region of the brain, has commonly been associated with motor control and balance [14], since it contains numerous neural circuits that are critical for motor learning and sensory processing. However, there is a growing consensus that it is also involved in social cognition, emotional regulation, and the modulation of social behavior. Studies have shown that damage to the cerebellum in humans can lead to deficits in social behavior, including problems with recognizing facial expressions, interpreting social cues, and processing emotional information [15]. Additionally, in humans, the cerebellum is believed to play a role in social communication, including speech and language perception [16]. Furthermore, recent research has suggested that the cerebellum may be involved in the regulation of social behavior, particularly in the context of empathy [17], and in recent years, researchers have come to agree that the cerebellum is vital for social cognition [18]. Hence, a better understanding of the cerebellum’s role in social behavior may also have important implications for understanding social skills in non-human animals [19,20,21,22]. 

Studies of mammals provide most of the information on the role of the cerebellum in emotion processing and social cognition. Despite the morphological differences between birds and mammals, the organization and the function of the avian cerebellum are remarkably similar to those of mammals [23,24]. The larger cerebellum observed in domesticated chickens may indicate that some of its functions are vital for adaptation to the selection pressures associated with living under the auspice of humans. 

A central feature in animal domestication of potential significance for coping with human handling is the concept of tameness, or reduced fear of humans. It has been suggested that tameness may not only be crucial for successful domestication but may actually drive large parts of the domestication syndrome [25]. When naturally shy and fearful farm foxes were selected for low fear of humans, they developed several traits associated with domesticated dog phenotypes in only a few generations, such as loss of pigmentation, shortening of legs and curly tails [26]. Selection of ancestral Red Junglefowl in a similar manner for low fear of humans caused the tame birds to develop a range of traits associated with domesticated chickens, such as increased growth rate, larger eggs, higher feed conversion, and reduced brain mass [25]. Furthermore, the intra-specific social behaviour also changed due to this selection [27], suggesting that tameness might be linked to different social dynamics through reduction of aggressive behaviours and increased social tolerance. Since the cerebellum plays a crucial part in social behaviour and emotional control, increased tameness may potentially be a key element underlying the enlarged cerebellum in chickens. 

Thus, here we aimed to analyse possible effects of increased tameness on brain composition in Red Junglefowl, divergently selected on tameness scoring over ten generations. This selection models the earliest phases of chicken domestication. We hypothesized that selection for tameness would be associated with reduced brain mass but increased relative mass of cerebellum. Furthermore, we hypothesized that this increase in cerebellum mass would be associated with an increased number of cerebellar neurons, potentially increasing the processing capacity of this part of the brain. 

## 2. Materials and Methods

### 2.1. Ethical Note

The experiments were carried out under the ethical licence from Linköping Animal Ethics Committee, licence no. 14916-2018. All procedures were carried out according to the protocol. 

### 2.2. Animals and Housing

The animals used in this study were Red Junglefowl from the tenth generation of selection. The first generation (S1) was created from an outbred population created by crossing two separate populations (one from a breeding station in Sweden, the other from Copenhagen Zoo) twice over two generations. The birds were selected for high fear of humans (HF) or low fear of humans (LF). In short, each bird’s behaviour during the standardized human approach test was graded on a scale from 1–5, with 1 being the tamest and 5 the most scared behaviour. The standardized fear-of-human-test was performed on the birds when they were 12 weeks old. It has been previously described in full how the breeding and selecting program works [28]. The populations were hatched, reared, and kept at the facility for ongoing research at Linköping University, where in the first 5 weeks they lived in the University’s hatchery on campus, and then moved to a research farm. Both lines have been kept together in the same environment. The layout of the birds’ home enclosures at the research farm consisted of an inside pen connected to an outside space (each measuring 3 m × 3 m). The indoor pen had free access to food, water, perches, nesting areas, and wood chip floor covering. The outside area had a gravel floor covering, a dust bath, and enhancing branches.

### 2.3. Brain Dissection and Assessment of Neurons

We extracted brains from 31 randomly selected birds from both the High-fear (N = 16, Females = 7, Males = 9) and Low-fear (N = 15, Females = 8, Males = 7) lines, all at the age of 33 months. The individuals were culled by rapid decapitation. For each bird, the sex and body weight were recorded just before the culling. The brains were extracted intact and immersion-fixed in 4% paraformaldehyde in 0.1 M phosphate buffer for three weeks. After tissue fixation, the brain weights were recorded (AE Adam PGW 453-e, precision of 0.001 g). We dissected and weighed the following brain regions: left and right cerebral hemispheres, left and right optic tectum, cerebellum, and the brain remainder (thalamus, remaining midbrain, and hindbrain). After dissection and weighing, the brain regions were stored in antifreeze solution (30% ethylene glycol, 30% glycerol, 40% phosphate buffer) at −18 °C. In this study, we focused on the whole brain, and the cerebellum mass and cellular composition, and included the brain remainder mass for comparison. 

We estimated the total number of cells in the cerebellum, divided into neurons and non-neuronal cells, by using the isotropic fractionator technique [29]. First, the cerebellum was transferred into a homogenizer (Tenbroeck tissue homogenizer) and mechanically dissociated in 40 mM sodium citrate with 1% Triton X-100 until there were no more tissue particles left in the solution. This process lasted 20 to 25 min and essentially transformed the cerebellum into a suspension of free cell nuclei. The total number of cells in the cerebellum was estimated by adding the fluorescent DNA marker 4′,6-Diamidine-2′-phenylindole dihydrochloride (DAPI) into the cell nuclei suspension. For each sample, four aliquots (10 μL) were counted using a Neubauer improved chamber and under a fluorescence microscope (Nikon eclipse 80i microscope, 400× magnification, numerical aperture 0.95). The coefficient of variation (CV) among the four aliquots were typically lower than 0.15. If the CV among the four aliquots was higher than 15%, two additional aliquots were counted. To distinguish between neurons and non-neuronal cells, we employed immunostaining technique. We used immunocytochemical detection of neuronal nuclear antigen NeuN, expressed in the nuclei of most neuronal types in the brain [30]. The samples were incubated overnight in a shaker and at 10 °C in mouse monoclonal antibody anti-NeuN 488 AlexaFluor conjugated (1:300 in phosphate-buffered saline; clone A60, Chemicon; MAB377X) [31,32]. A minimum of 500 nuclei were counted to estimate the proportion of neurons in the sample. By subtracting the number of neurons from the total cell count, we determined the number of non-neuronal cells [29,31].

### 2.4. Variables 

The absolute masses of the whole brain and dissected regions were obtained directly from weighing. The relative brain mass was then obtained by dividing the weight of the total brain by the body weight of each individual. The relative mass of each brain region was obtained by dividing its weight with the total brain mass. The number of neurons and of non-neural cells in the entire cerebellum was calculated by extrapolating the average numbers counted in the 10 μL samples to the entire volume of the cerebellar suspension. The neuronal and non-neuronal density were obtained by dividing the number of neurons or non-neuronal cells in the cerebellum with the weight of the cerebellum.

### 2.5. Statistics

The difference in brain mass, absolute and relative mass of the cerebellum and brain remainder, number of neurons, number of non-neurons, neuron density, and non-neuron cell density were compared between the two selection lines and the sexes using generalized linear models (GLM), with probability distribution normal and identity link function. The link function was used since the data inspection showed that no transformation of data was necessary before significance testing. The model included the predictor variables of line and sex and the interaction between the two. We only report interactions when these were found to be significant.

## 3. Results

**Figure 1:**
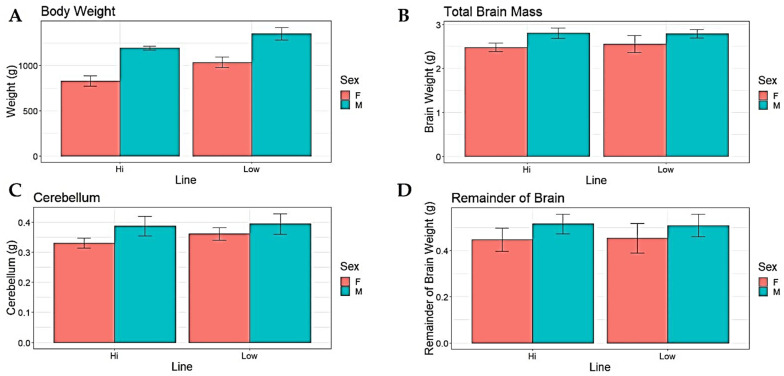
Average values (+/−SEM) of absolute mass of total body, brain, brainstem, and cerebellum in Red Junglefowl females (F) and males (M) selected for high (HF) vs. low fear (LF) of humans. (**A**) Body mass, (**B**) total brain, (**C**) cerebellum, (**D**) remainder of brain.

Both line and sex had a significant effect on body weight, where birds from the low fear line (LF) had higher body weight than those from the high fear line (HF), and in both lines, males weighed more than females (Figure 1A, Table 1). Sex also had a significant effect on total brain mass and remainder of brain mass, both being bigger in males, whereas there were no effects of line on either (Figure 1B,C, Table 1). LF birds had larger cerebellum mass, and within both lines the males had larger cerebellum size compared to the females (Figure 1D, Table 1). 

**Figure 2:**
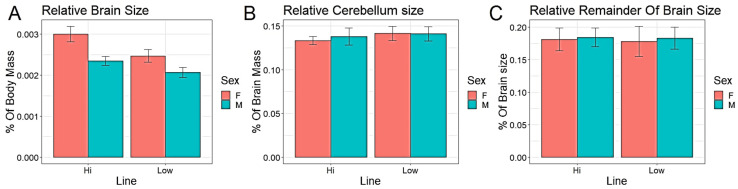
(**A**) Relative brain size in % of body mass (mean ± SE), (**B**) relative cerebellum size in % of total brain mass (mean ± SE), and (**C**) the relative mass of the brain remainder in % of total brain mass (mean ± SE).

Since both body weight and total brain size differed significantly between lines and sexes, we further analysed the relative sizes of the brain and its parts. The relative brain mass (% of total body weight) was significantly larger in HF compared to LF and in females compared to males (Figure 2A, Table 1). Furthermore, there was a significant interaction between line and sex, caused by the effect of line being larger in females (Wald χ^2^ = 6.53, df = 1, *p* < 0.05). The relative cerebellum mass (% of total brain mass) was larger in LF, whilst there was no effect of sex (Figure 2B, Table 1). The relative mass of the brain remainder did not differ between the selection lines or between sexes (Figure 2C, Table 1).

**Figure 3:**
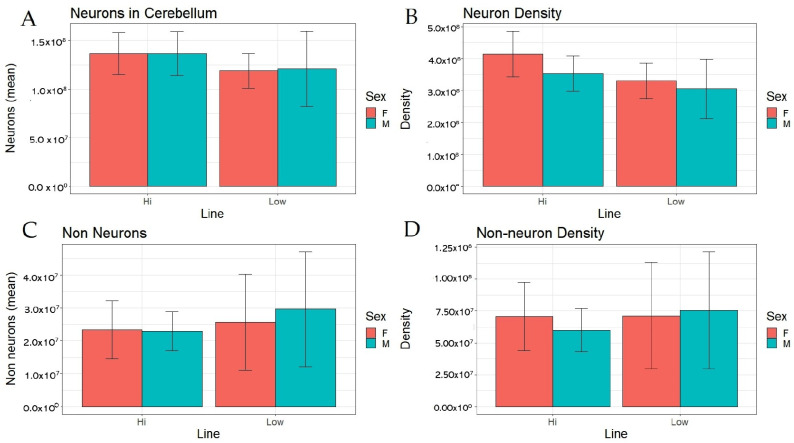
Absolute count and density of neurons and non-neurons in cerebellum in female (F) and male (M) Red Junglefowl selected for high (HF) or low (HF) fear of humans. (**A**): Number of neurons; (**B**): Neuron density; (**C**): Number of non-neurons; (**D**): Non-neuron density.

To further analyse differences in brain composition between the lines, we estimated numbers of neurons and non-neurons in cerebellum. HF birds tended to have more neurons in absolute numbers, and they had significantly higher cerebellar neuron density (number of neurons per mg cerebellum tissue) (Figure 3A,B, Table 1), whilst there were no effects of sex on these variables. However, there were no significant differences in absolute numbers or density of non-neurons between the lines or any effect of sex (Figure 3C,D, Table 1).

## 4. Discussion

The aim of our study was to investigate how brain size, neuron density, and cerebellum size is affected by selection for low or high fear of humans in Red Junglefowl (RJF), as a proxy for early domestication responses. The results show significant effects on body weight, as well as on absolute and relative sizes of the whole brain and of the cerebellum. Furthermore, number and density of neurons in cerebellum was significantly affected by the selection. The findings regarding size effects mirror previously reported differences between contemporary domesticated chickens and ancestral Red Junglefowl, suggesting that domestication effects on the size and composition of brain and cerebellum may have been driven by reduced fearfulness towards humans in the early phases of domestication. 

The domestication of chickens from Red Junglefowl in South East Asia likely occurred around 8000 years ago [9], though recent studies suggest a relatively more recent period of domestication [10]. Although the driving forces behind this process remain speculative, it is inevitable that reduced fear of humans (increased tameness) must have been a central aspect of the initial domestication responses, not least since RJF in the wild is a highly fearful and shy species [33]. Farm foxes selected for low fear of humans as a model for early domestication developed a suite of phenotypic traits associated with the domestication syndrome in a few generations [26], which suggests that this syndrome may be driven by reduced fear. Similar effects have previously been reported by us for Red Junglefowl selected for increased tameness, where these birds developed a range of phenotypic changes commonly associated with domesticated chickens, such as increased growth, larger eggs, increased feed conversion, and modified social behaviour [25,27]. One of the main aspects of the domestication syndrome is a reduction in relative brain size, which appears in most species [34]. In chickens, absolute brain size has increased in modern domesticated egg-layers, but relative to body size, the brain has become smaller [11]. However, as shown by Henriksen et al. [11], this does not pertain to all brain parts similarly, since the cerebellum in domesticated chickens has actually grown in size relative to the rest of the brain. It should be noted that LF birds are more prone to feed and have a higher feed efficiency (growth per g feed intake) [35], which may drive the body size difference between the lines. Although we have no data on the actual feed intake, it is clear that LF birds appear to consume more feed. 

Our present study demonstrates that selection for tameness affects not only body weight, but also changes the relative mass of the brain and the absolute size of the cerebellum, as well as relative cerebellum size, in the same direction as that seen in contemporary domesticated chickens. As shown in Figure 1D and Figure 2C, there were no significant line effects on the absolute or relative sizes of the remainder of the brain, indicating that the observed selection responses are specific to the cerebellum and not applicable to the entire brain. The fact that the brain remainder was not affected by selection in the same way strongly suggests that this was specific to the cerebellum, indicating that the cerebellum probably fills an important function in tame birds. This is consistent with the idea that tameness may also have been the driving force underlying this aspect of the domesticated phenotype in chickens. However, although the more fearful line of birds had a smaller cerebellum relative to total brain mass, the neuron density was in fact higher, contrary to our hypotheses. This is not in line with comparisons between present-day domesticated egg layers and ancestral Red Junglefowl that found a higher number of neurons in the larger cerebellum of the domesticates [36], which in turn is consistent with studies of mice which have shown that increased number of cerebellar neurons is related to a decrease in fear-related behaviour [37]. Of course, neural processing capacity is a function of both the number of neurons and synaptic networks, and in this study, we were not able to estimate synapse densities. It is noteworthy that the size of the cerebellum relative to the rest of the brain increases substantially during the first four weeks of life of chicks, unlike all other brain parts, indicating that ontogenetic factors may play an important role in the development of this part of the brain [36]. In rats, extensive synaptogenesis occurs in the cerebellum during the early neonatal period [38], and for future studies, it will be important to include this aspect in comparisons such as ours.

The cerebellum was previously considered to mainly be a motor control center of the brain, but its function has lately been fundamentally reconsidered [15]. A plethora of research in different species shows that it is involved in various social processes, as well as in emotional fear learning and fear extinction [39]. Hence, the cerebellum is a key aspect of the brain’s “fear network”, together with the amygdala, prefrontal cortex, and hippocampus. The cerebellar nuclei are connected to these other brain parts and essential to this fear network [40]. The fastigial nucleus is a part of the cerebellum that projects to the thalamus, a brain region involved in emotion regulation. A recent study of male mice found that inhibition of certain signals from the fastigial nucleus in the cerebellum to the thalamus resulted in impaired extinction learning, and an increased expression of fear behaviour [41]. A previous study of brain composition and its relationship to behavioural effects in Red Junglefowl used an intercross of the high and low fear lines studied by us [20]. In such an intercross, phenotypic differences between the purebred lines segregate, and it was found that birds with larger cerebellum mass had reduced reactions towards a fearful but harmless stimulus over time, indicating a better fear memory consolidation. This would appear to be an important aspect of tameability and hence highly relevant for the early domestication process. It is also important to note that sex differences play a crucial role in the social behaviour of this species. The sex effect is essential to consider when studying the social behaviour of any animal species when utilizing them as models to study social behaviour. For instance, a study involving mice demonstrated that individually housed females exhibited reduced exploration and increased anxiety compared to group-housed females, whereas individually housed males showed the opposite pattern. Findings like these suggest that selection for tameness may have distinct effects on the sexes [42]. 

In conclusion, Red Junglefowl (RJF) selected for reduced fear of humans (LF) had smaller brains that RJF selected for increased fear in relative measures. Furthermore, LF had larger cerebellum mass in relation to the size of the rest of the brain, mirroring previously known differences between contemporary domesticated chickens and ancestral RJF. However, HF had significantly higher neuron density in the cerebellum, contrary to what has been found in comparisons between modern chickens and RJF. The cerebellum plays a key role in fear learning and fear extinction, which is essential in tameness and domestication. Our results therefore suggest that selection for increased tameness may have driven some of the changes in brain composition that have previously been reported for domesticated chickens.

## Figures and Tables

**Table 1:** The statistical analyses and the results performed on the selected Red Junglefowl showing the significance of line and sex.

Dependent Variable	Line (Wald χ^2^)	Sex (Wald χ^2^)
Body Weight	103.921 ***	360.274 ***
Absolute Brain Size	0.485	38.179 ***
Absolute Cerebellum Size	4.250 *	23.838 ***
Remainder of Brain Absolute Size	0.001	12.220 ***
Number of Neurons	3.687	0.013
Number of Non-neurons	1.185	0.190
Neuron Density	8.010 **	3.366
Non-neuron Density	0.499	0.080
Relative Brain Size	70.613 ***	118.531 ***
Relative Cerebellum Size	4.363 *	0.556
Relative Remainder of Brain Size	0.977	0.972

Significant codes: *** = 0.001, ** = 0.01, * = 0.05.

## Data Availability

The full dataset is available as Supplementary Table S1.

## Supplementary Materials

The following supporting information can be downloaded at: https://www.mdpi.com/2076-3425/13/7/988/s1, Table S1: Complete data set.
