# Correction: Gjøen et al. Selection for Reduced Fear of Humans Changes Brain and Cerebellum Size in Red Junglefowl in Line with Effects of Chicken Domestication. *Brain Sci.* 2023, *13*, 988

**DOI:** 10.3390/brainsci13101465

**Published:** 2023-10-17

**Authors:** Johanna Gjøen, Felipe Cunha, Per Jensen

**Affiliations:** AVIAN Behavioural Physiology and Genomics Group, IFM Biology, Linköping University, 58183 Linköping, Sweden; johanna.gjoen@liu.se (J.G.); felipebrcunha@gmail.com (F.C.)

## Text Correction

There were errors in the original publication [1]:

1. In the Abstract, the following sentence is wrong: “Our analysis revealed that birds selected for low fear of humans (LF) had larger absolute brain mass, but smaller relative brain mass, compared to those selected for high fear of humans (HF)”.

The correct wording should be “Our analysis revealed that birds selected for low fear of humans (LF) had smaller relative brain mass compared to those selected for high fear of humans (HF)”.

2. Furthermore, in the last para of the Discussion, the starting sentence is wrong: “In conclusion, Red Junglefowl (RJF) selected for reduced fear of humans (LF) had larger brains that RJF selected for increased fear, both in relative and absolute measures”.

Instead, it should read as “In conclusion, Red Junglefowl (RJF) selected for reduced fear of humans (LF) had smaller brains that RJF selected for increased fear in relative measures”.

The authors state that the scientific conclusions are unaffected. These corrections were approved by the Academic Editor. The original publication has also been updated.
